# Supplementary material for: The Identification of Subphenotypes and Associations with Health Outcomes in Patients with Opioid-Related Emergency Department Encounters Using Latent Class Analysis
Source: Int J Environ Res Public Health. 2022 Jul 21;19(14):8882. doi: 10.3390/ijerph19148882 (PMC9321801; doi:10.3390/ijerph19148882)
Supplement: Supplementary file 1 [file ijerph-19-08882-s001.zip › Table S1.pdf]

Table S1. ICD-10-CM diagnosis codes defining opioid-related encounters.

Type of opioid-related condition	ICD-10-CM diagnosis codes
Abuse or dependence	F11 series: Opioid-related disorders (except F11.21)
Adverse event	T40.0X5: Adverse effect of opium<br>T40.2X5: Adverse effect of other opioids<br>T40.3X5: Adverse effect of methadone<br>T40.4X5: Adverse effect of other synthetic narcotics<br>T40.605: Adverse effect of unspecified narcotics<br>T40.695: Adverse effect of other narcotics
Poisoning, including self-harm	T40.0X1, 0X2, 0X3, 0X4: Poisoning by opium–accidental, intentional self-harm, assault, or undetermined<br>T40.1X1, 1X2, 1X3, 1X4: Poisoning by heroin–accidental, intentional self-harm, assault, or undetermined<br>T40.2X1, 2X2, 2X3, 2X4: Poisoning by other opioids–accidental, intentional self-harm, assault, or undetermined<br>T40.3X1, 3X2, 3X3, 3X4: Poisoning by methadone–accidental, intentional self-harm, assault, or undetermined<br>T40.4X1, 4X2, 4X3, 4X4: Poisoning by other synthetic narcotics–accidental, intentional self-harm, assault, or undetermined<br>T40.601–T40.604: Poisoning by unspecified narcotics–accidental, intentional self-harm, assault, or undetermined<br>T40.691–T40.694: Poisoning by other narcotics–accidental, intentional self-harm, assault, or undetermined
