# Supplementary material for: The Identification of Subphenotypes and Associations with Health Outcomes in Patients with Opioid-Related Emergency Department Encounters Using Latent Class Analysis
Source: Int J Environ Res Public Health. 2022 Jul 21;19(14):8882. doi: 10.3390/ijerph19148882 (PMC9321801; doi:10.3390/ijerph19148882)
Supplement: Supplementary file 1 [file ijerph-19-08882-s001.zip › Table S2.pdf]

Table S2. ICD-10 codes.

Chronic Pain:

From the Agency for Healthcare Research and Quality (chronic primary pain, psychogenic pain, chronic postsurgical and posttraumatic pain, and chronic neuropathic pain were included. Additional codes were included for chronic secondary musculoskeletal pain and chronic secondary visceral pain. Codes were excluded for acute pain, chronic cancer- related pain, and chronic secondary headache or orofacial pain)

'F45.4', 'G89.0', 'G89.2', 'G89.21', 'G89.22', 'G89.28', 'G89.29', 'G89.4', 'M08.1', 'M25.50', 'M25.51', 'M25.55', 'M25.56', 'M25.57', 'M43.2', 'M43.3', 'M43.4', 'M43.5', 'M43.6', 'M45', 'M46.1', 'M46.3', 'M46.4', 'M46.9', 'M47', 'M48.0', 'M48.1', 'M48.8', 'M48.9', 'M50.8', 'M50.9', 'M51', 'M53.1', 'M53.2', 'M53.3', 'M53.8', 'M53.9', 'M54', 'M60.8', 'M60.9', 'M63.3', 'M79.0', 'M79.1', 'M79.2', 'M79.6', 'M79.7', 'M96.1'

Alcohol use disorders (Hoffman 2015):

‘F10.1’, ‘F10.2’, ‘F10.3’, ‘F10.9’ excluding F10.11 and F10.21 (in remission)

Psychoses:
‘F20’, ‘F21’, ‘F22’, ‘F23’, ‘F24’, ‘F25’, ‘F28’, ‘F29’

Depression:
‘F32’, ‘F33’

Liver disease:
‘K70’, ‘K71’, ‘K72’, ‘K73’, ‘K74’, ‘K75’, ‘K76’, ‘K77’

Pregnancy:
‘O00’-‘O9A’

Cocaine use:
‘F14’ excluding ‘F14.11’ and ‘F14.21’ (in remission)

Amphetamine use (Ridley 2015):
‘F15.0’, ‘F15.1’, F15.2’, ‘F15.3’, ‘F15.4’, ‘F15.5’, F15.6’, ‘F15.7’, ‘F15.8’, ‘F15.9’
