# Supplementary material for: The Identification of Subphenotypes and Associations with Health Outcomes in Patients with Opioid-Related Emergency Department Encounters Using Latent Class Analysis
Source: Int J Environ Res Public Health. 2022 Jul 21;19(14):8882. doi: 10.3390/ijerph19148882 (PMC9321801; doi:10.3390/ijerph19148882)
Supplement: Supplementary file 1 [file ijerph-19-08882-s001.zip › Table S4.pdf]

**Table S4.** Patient characteristics odds ratios by latent class.

| | Class 1 Chronic Pain | Class 2 Alcohol Use | Class 3 Depression & Pain | Class 4 Psychosis, Liver Disease, & Polysubstance Use | Class 5 Pregnancy |
|---|---|---|---|---|---|
| Age | ref | 1.00 | 1.00 | 1.00 | 0.92 |
| Sex | | | | | |
| Female | ref | 0.29 | 1.44 | 0.53 | N/A |
| Male | | | | | |
| Payer | | | | | |
| Medicare | ref | ref | ref | ref | ref |
| Medicaid | ref | 2.07 | 0.70 | 1.19 | 50 |
| Private | ref | 1.28 | 0.60 | 0.59 | 33.33 |
| Self-pay | ref | 2.49 | 0.40 | 1.05 | 20 |
| No charge | ref | 3.98 | 0.48 | 1.43 | 25 |
| Other | ref | 1.34 | 0.45 | 0.59 | 20 |
| Median income | | | | | |
| Top quartile | ref | ref | ref | ref | ref |
| 2nd quartile | ref | 0.97 | 1.12 | 0.89 | 0.91 |
| 3rd quartile | ref | 1.13 | 1.24 | 0.93 | 0.88 |
| 4th quartile | ref | 1.45 | 1.40 | 1.00 | 0.79 |
| Urbanicity | | | | | |
| Central metropolitan | ref | ref | ref | ref | ref |
| Fringe metropolitan | ref | 0.94 | 1.25 | 0.85 | 0.72 |
| 250-999K | ref | 0.80 | 1.29 | 0.79 | 0.66 |
| 50-250K | ref | 0.80 | 1.15 | 0.70 | 0.57 |
| Micropolitan | ref | 0.61 | 1.20 | 0.65 | 0.53 |
| Non-core | ref | 0.56 | 1.09 | 0.61 | 0.49 |
