# Supplementary figures and images for: The Identification of Subphenotypes and Associations with Health Outcomes in Patients with Opioid-Related Emergency Department Encounters Using Latent Class Analysis
Source: Int J Environ Res Public Health. 2022 Jul 21;19(14):8882. doi: 10.3390/ijerph19148882 (PMC9321801; doi:10.3390/ijerph19148882)

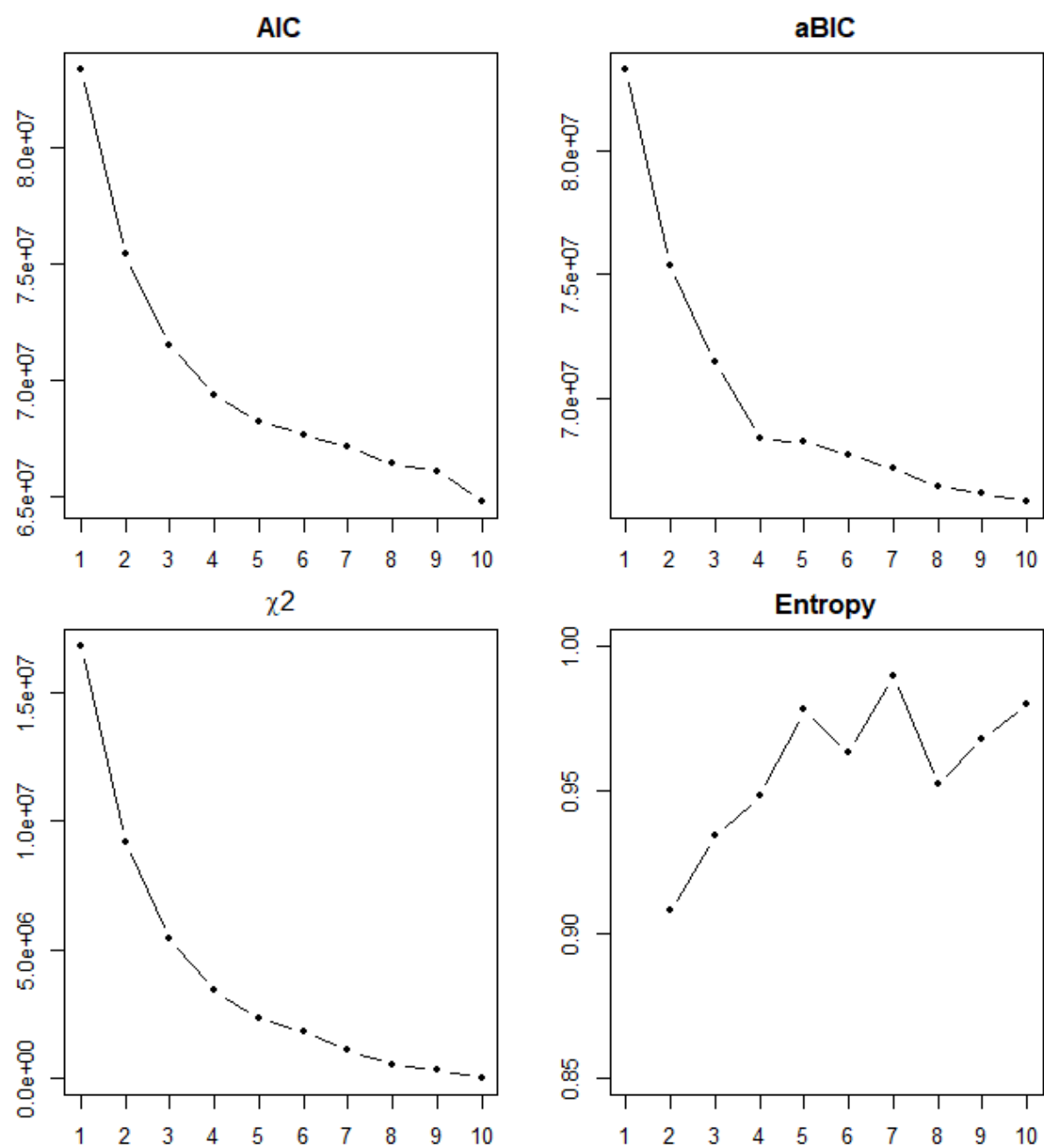

Figure S1. Information criteria-based metrics by number of classes in model.

Supplement: Supplementary file 1 [file ijerph-19-08882-s001.zip › Figure S1.pdf]
